# Systemic lupus erythematosus therapeutic strategy: From immunotherapy to gut microbiota modulation

**DOI:** 10.7555/JBR.38.20240009

**Published:** 2024-05-31

**Authors:** Vitaly Chasov, Ekaterina Zmievskaya, Irina Ganeeva, Elvina Gilyazova, Damir Davletshin, Maria Filimonova, Aygul Valiullina, Anna Kudriaeva, Emil Bulatov

**Affiliations:** 1 Institute of Fundamental Medicine and Biology, Kazan Federal University, Kazan 420008, Russia; 2 Shemyakin-Ovchinnikov Institute of Bioorganic Chemistry, Russian Academy of Sciences, Moscow 117997, Russia

**Keywords:** systemic lupus erythematosus, immunotherapy, monoclonal antibodies, bispecific antibodies, chimeric antigen receptor T cell, fecal microbiota transplantation

## Abstract

Systemic lupus erythematosus (SLE) is characterized by a systemic dysfunction of both the innate and adaptive immune systems, leading to an attack on healthy tissues of the body. During the development of SLE, pathogenic features, such as the formation of autoantibodies against self-nuclear antigens, cause tissue damage including necrosis and fibrosis, with increased expression levels of the type Ⅰ interferon-regulated genes. Standard treatments for lupus with immunosuppressants and glucocorticoids are not effective enough but cause side effects. As an alternative, more effective immunotherapies have been developed, including monoclonal and bispecific antibodies that target B cells, T cells, co-stimulatory molecules, cytokines or their receptors, and signaling molecules. Encouraging results have been observed in clinical trials with some of these therapies. Furthermore, a chimeric antigen receptor T cell therapy has emerged as the most effective, safe, and promising treatment option for SLE, as demonstrated by successful pilot studies. Additionally, some emerging evidence suggests that gut microbiota dysbiosis may significantly contribute to the severity of SLE, and the normalization of the gut microbiota through methods such as fecal microbiota transplantation presents new opportunities for effective treatment of SLE.

## Introduction

Systemic lupus erythematosus (SLE) is a typical autoimmune disease in which the overactive immune system attacks healthy tissues, causing multiple lesions and chronic inflammation of the organs ^[ 1] ^. The disease is known to develop under the influence of genetic, epigenetic, and environmental factors that cause immune dysregulation and the formation of autoantibodies in the blood, which target nucleic acids and their related proteins. Lupus is highly heterogeneous, affecting almost all major organs and tissues in humans, including the skin, joints, kidneys, lungs, heart, and blood cells ^[ 2] ^.

Because traditional treatments, such as glucocorticoids and immunosuppressants, have a limited efficacy and cause side effects in many patients, the targeted therapy is becoming an increasingly popular treatment option ^[ 3– 4] ^. Since the US Food and Drug Administration (FDA) approved the first biologic for the treatment of patients with active SLE in 2011, new biologic therapies with varying mechanisms of action have been continuously developed and tested for safety and clinical efficacy, including monoclonal antibodies (mAbs) and bispecific antibodies (BsAbs) that may act on primary targets, such as B and T lymphocytes, co-stimulatory molecules, cytokines or their receptors ^[ 5– 8] ^. In addition, therapies for systemic autoimmune diseases (SAIDs), of which SLE is a representative one, not only suppress the activity of various autoreactive immune clones of B and T cells and their receptors using antibodies but also present the possibility of therapeutic use of chimeric antigen receptor (CAR)-T cells ^[ 9] ^. An increased expectation for breakthroughs in the treatment of SAIDs is directly linked to recent successes of the CAR-T therapy in the treatment of systemic lupus and systemic sclerosis (SSc) ^[ 10– 11] ^.

Some investigators believe that, in addition to environmental and genetic factors, the microbiota is also responsible for the pathogenesis of systemic lupus, although the mechanisms of its influence are not yet fully understood ^[ 12] ^. Specifically, a pilot study reported that human microbiota imbalance in SLE was correlated with immunological characteristics of the patients, such as serum C4 levels, suggesting that normalization of the microbiota may restore dysregulated immunity in SLE patients ^[ 13] ^. Indicators of immune system health, such as impaired T follicular helper (Tfh)/T follicular regulatory (Tfr) and T helper 17 (Th17)/regulatory T (Treg) cell ratios and levels of inflammatory cytokines in mice and SLE patients, were also found to be associated with changes in gut microbiota composition ^[ 12] ^. All these data suggest intestinal dysbiosis as an important element in the development of SLE, and efforts aimed at its normalization are quite suitable for the role of a new means of treating this disease. Pan *et al* ^[ 14]^ reported that nutritional therapies, antibiotics, vaccination, probiotics or prebiotics, and fecal microbiota transplantation (FMT) might be used to influence intestinal dysbiosis. For example, FMT has been shown to be a safe, feasible, and potentially effective approach for modifying the gut microbiota and its metabolic profile, which is considered a promising SLE therapy ^[ 15– 16] ^.

The present review discusses current advances in immunotherapies with targeted biological agents and future research in these areas for successful treatment of SLE. We also summarize new insights into the mechanisms by which microbiota dysbiosis influences the pathogenesis of SLE and promising therapeutic strategies that target the gut microbiota to cure this disease.

## Immunotherapies of SLE

SLE is a chronic disease in which systemic inflammation affects organs and tissues throughout the body. The underlying immune system dysfunction causes damage to the organs and tissues because of the formation of autoantibodies. Most patients suffer from chronic joint pain, as well as frequent circulatory, renal, and cardiovascular dysfunction, which may be life-threatening and lead to death during exacerbations ^[ 2, 17] ^.

Because of the abnormal activation of B and T lymphocytes, the course of SLE is accompanied by the production of antinuclear antibodies, although the exact pathogenesis of this disease remains unclear. According to the available data, a combination of environmental and hereditary factors plays a role in the etiology ^[ 18] ^. While glucocorticoids and immunosuppressants have been the first-line therapy for SLE for many decades and are quite effective in many cases, they are known to be addictive and cause significant side effects ^[ 19– 20] ^. Therefore, the targeted therapy using biological agents has recently become a more promising option ^[ 3– 4] ^. These novel biological agents have different mechanisms of action and effectively target B cells, T cells, T cell co-stimulatory molecules, cytokines, their receptors, and other molecular targets. The current review mainly focuses on mAbs as biological agents, although BsAbs are also actively being developed and used in the treatment of patients with SLE ( ***Table 1***) ^[ 4] ^.

**Table 1:** Summary of biological drugs for treating systemic lupus erythematosus under clinical trials

Drugs	Mechanism of action	Clinical trial phase
B cell activity inhibitors		
Rituximab	Chimeric anti-CD20 mAb, eliminates B cells and a subset of T cells	Phase Ⅳ
Belimumab	Anti-BAFF, IgG1 mAb that specifically binds to the soluble form of human B-cell-stimulating protein	Phase Ⅳ
Blisibimod	Anti-BAFF fusion protein consisting of four BAFF binding domains	Phase Ⅲ
Obexelimab (XmAb5871)	mAb, targeting CD19 and FcγRⅡb on B cells, NCT02725515	Phase Ⅱ
Epratuzumab	Anti-CD22 human mAb	Phase Ⅲ
Mosunetuzumab	BsAb, binds CD20 and CD3 to engage T cells	Phase Ⅰ
PRV-3279	Fv specific BsAb, simultaneously ligates the inhibitory molecule CD32B (FcγRⅡb) with the B-cell receptor component CD79B on B cells	Phase Ⅱ
Rozibafusp alfa (AMG-570)	BsAb, targeting ICOSL and BAFF	Phase Ⅱ
Ianalumab	mAb, anti-BAFFR	Phase Ⅲ
Obinutuzumab	Human anti-CD20 mAb	Phase Ⅲ
Telitacicept	Fully human TACI-Fc fusion protein that targets BLyS and APRIL	Phase Ⅳ
Atacicept	Recombinant fusion protein, anti-BLyS and APRIL	Phase Ⅲ
Tabalumab	Anti-BAFF mAb	Phase Ⅲ
Ocrelizumab	Anti-CD20 mAb	Phase Ⅲ
Abetimus	It is made of four double-stranded oligodeoxyribonucleotides that are attached to a carrier platform and are designed to block specific B-cell anti-double-stranded DNA antibodies	Phase Ⅲ
T cell or T cell co-stimulator blockers		
Abatacept	Protein, CTLA4-Ig construct, and CD80/86 costimulation inhibitor, selectively modulates a key co-stimulatory signal for the full activation of T lymphocytes expressing CD28. It inhibits activated T cells and antigen-presenting cells	Phase Ⅲ
Itolizumab	Anti-CD6 mAb	Phase Ⅰ
Iscalimab	Anti-CD40 mAb	Phase Ⅱ
Milatuzumab	Anti-CD74 mAb	Phase Ⅰ
Lulizumab pegol	Anti-CD28 mAb	Phase Ⅱ
Acazicolcept	Anti-CD28 and ICOS BsAb	Phase Ⅱ
LY3361237	Agonist mAb to the checkpoint inhibitory receptor BTLA (B and T lymphocyte attenuator)	Phase Ⅱ
Dazodalibep (VIB4920)	Anti-CD40 ligand-Tn3 fusion protein	Phase Ⅱ
Dapirolizumab pegol	Anti-CD40L mAb	Phase Ⅲ
BI655064	Anti-CD40 mAb	Phase Ⅱ
Targeting cytokine or cytokine receptor		
Infliximab	Chimeric human-mouse anti-(TNFα) mAb	Phase Ⅱ/Ⅲ
Etanercept	It is a fusion protein produced by recombinant DNA, TNF inhibitor	Phase Ⅱ
Anifrolumab	mAb, anti-IFNα receptor	Phase Ⅲ Approved by FDA in 2021
Sifalimumab	Anti-IFNα mAb	Phase Ⅱ
Rontalizumab	Anti-IFNα mAb	Phase Ⅱ
JNJ-55920839	Anti-IFNα mAb	Phase Ⅰ
IFN-α kinoid	A therapeutic vaccine composed of IFNα2b coupled to a carrier protein	Phase Ⅱ
BT-063	Anti-IL-10 mAb	Phase Ⅱ
Ustekinumab	MAb that binds to the p-40 subunit of both IL-12 and IL-23 so that they subsequently cannot bind to their receptors to activate T cells	Phase Ⅲ
Secukinumab	Anti-IL17A mAb	Phase Ⅲ
Guselkumab	Anti-IL23 mAb	Phase Ⅱ
Vunakizumab (SHR1314)	Anti-IL17A mAb	Phase Ⅱ
Avizakimab (BOS161721)	Anti-IL21 mAb	Phase Ⅱ
Tocilizumab	mAb against IL-6R, inhibits B cell differentiation, M2 macrophages, TH17 polarization, and myofibroblast activation	Phase Ⅰ
ALX-0061	Anti-IL-6R BsAb	Phase Ⅱ
MRA (MRA003)	Anti-IL-6 mAb	Phase Ⅰ
Drugs targeting pDCs		
Litifilimab	Anti-BDCA2 mAb	Phase Ⅲ
Daxdilimab	Anti-ILT-7 mAb, which specifically induces the depletion of pDCs	Phase Ⅱ
Abbreviations: APRIL, a proliferation-inducing ligand; BAFF, B-cell activating factor; BAFFR, BAFF-receptor; BDCA2, blood dendritic cell antigen 2; BLyS, B lymphocyte stimulator; BsAb, bispecific antibody; BTLA, B and T lymphocyte attenuator; CD, cluster of differentiation; CTLA-4, cytotoxic T-lymphocyte-associated protein 4; FcγRⅡb, Fc gamma receptor Ⅱb; ICOSL, inducible T-cell costimulator ligand; IFN, interferon; IL, interleukin; ILT, immunoglobulin-like transcript; TACI, transmembrane activator and calcium modulator and cyclophilin ligand interactor; TNF, tumor necrosis factor; pDCs, plasmacytoid dendritic cells.		

### B cell activity inhibitors

**Figure 1:**
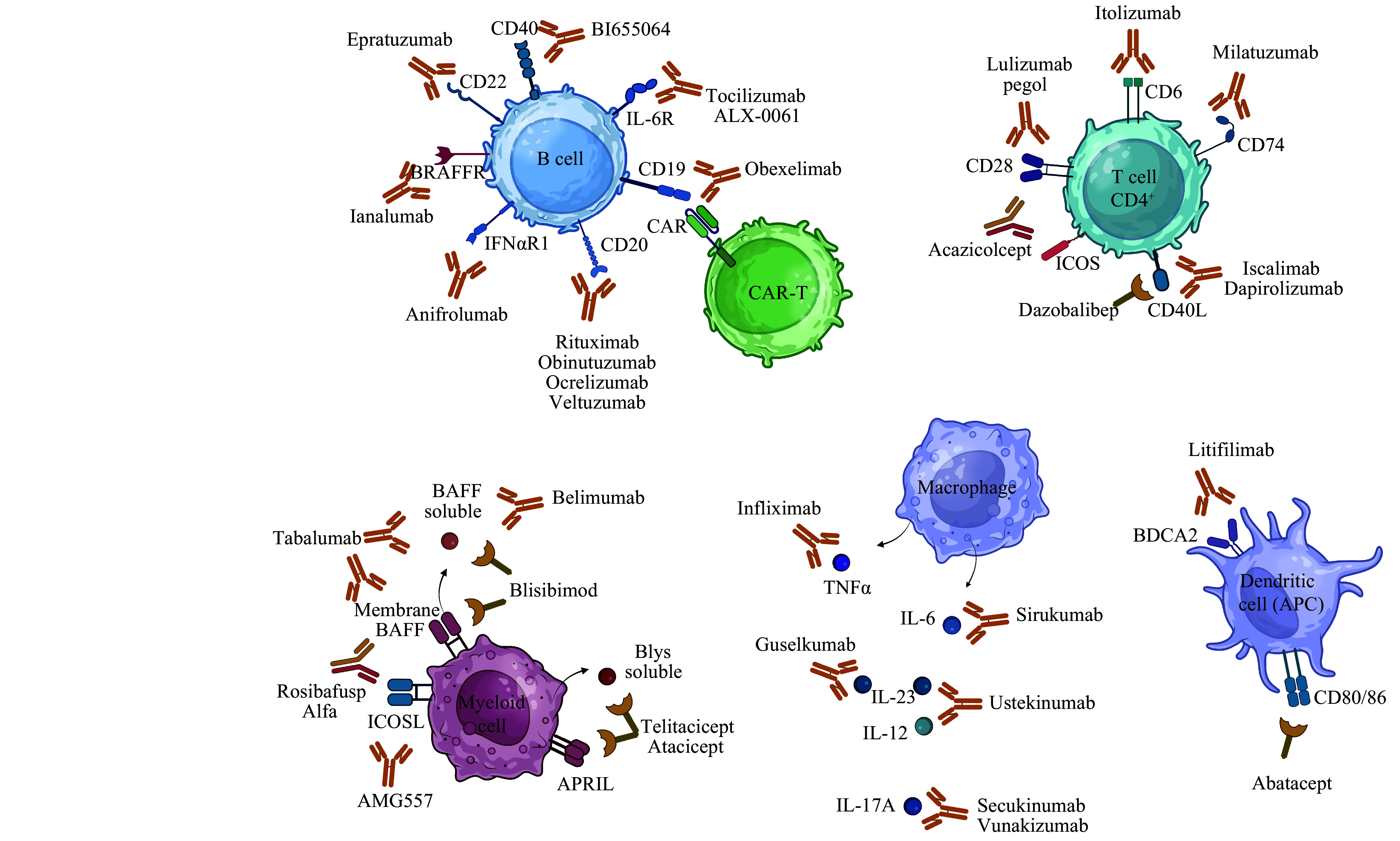
Targeted therapy of systemic lupus erythematosus. The figure shows the sites of action using biological agents with a focus on therapeutic monoclonal antibodies and CAR-T cells. Abbreviations: APC, antigen-presenting cell; APRIL, a proliferation-inducing ligand; BAFF, B-cell activating factor; BDCA2, blood dendritic cell antigen 2; CAR-T cell, chimeric antigen receptor T cell; ICOS, inducible T-cell costimulator; ICOSL, inducible T-cell costimulator ligand; TNF, tumor necrosis factor.

The presence of unique receptors on the membranes of B lymphocytes enables them to recognize foreign proteins, or antigens, and help the body produce antibodies specifically designed to fight them ^[ 21] ^. When autoantibodies target the body's proteins, tissue damage and inflammation occur. B cells not only produce autoantibodies but also release cytokines and, *via* their antigen-presenting role, facilitate the activation and development of autoreactive T cells ^[ 22] ^. These characteristics mean that the B-cell activity plays a major role in determining the pathophysiology of SLE. The primary goal of targeted treatment of SLE in this setting is the development of biologics that inhibit B-cell-related receptors to prevent the activation of cytokines and signaling molecules ^[ 4] ^. B cell maturation, differentiation, autoimmunity, and antibody production are controlled by B-cell activating factor of the tumor necrosis factor family (BAFF) produced by myeloid cells, among others ( ***Fig. 1***). When BAFF binds to three receptors ( *i.e.*, BAFF-receptor, transmembrane activator and calcium modulator and cyclophilin ligand interactor, and B-cell maturation antigen), B-cell eradication is disrupted ^[ 23– 24] ^. For example, an elevated level of BAFF has been found to be associated with a high activity of SLE ^[ 24] ^. Another protein in this family, a proliferation-inducing ligand (APRIL), also stimulates BAFF-like activity after combining with BAFF to form heterotrimers ^[ 25– 26] ^.

The FDA has approved belimumab as the first biologic treatment for SLE. BAFF cannot bind to any of the three receptors to which when bound by this mAb ^[ 27] ^. Two major phase Ⅲ clinical trials established the efficacy of this antibody in active seropositive individuals with minimal complement and positive antibodies to dsDNA ^[ 28– 29] ^. Belimumab is currently in phase Ⅳ clinical trials ( ***Table 1***). Other drugs targeting the BAFF/APRIL family are also available ( ***Fig. 1*** and ***Table 1***).

CD20, whose function is to link B cell receptor (BCR) signaling to promote interactions in the microenvironment, is known as a calcium channel in the cell membrane and is expressed on the surface of all B cells ^[ 30– 31] ^. CD22 is a transmembrane inhibitory protein whose function is to inhibit BCR signaling, a regulatory molecule that plays an important role in B cell activation ^[ 32– 34] ^. CD20 and CD22 receptors are major targets of biological agents in the immunotherapy ( ***Fig. 1***) ^[ 33, 35– 36] ^. A phase Ⅳ clinical trial is currently underway for rituximab, an anti-CD20 mAb. Adding belimumab after rituximab significantly lowered the risk of severe flare-ups and serum antibody levels to dsDNA in a special SLE therapy trial ^[ 37] ^. This suggests that the combination of drugs may be a good way to treat the disease.

CD19 is another transmembrane protein that functions as a co-receptor for BCR and stimulates B cell growth, activation, proliferation, and signaling. One study showed that mitogen-activated protein kinase function, calcium release, and B cell proliferation were all enhanced, when CD19 and BCR were bound ^[ 38] ^. The Fc gamma receptor Ⅱb (FcγRⅡb) on the surface of B cells has been shown to have some affinity for IgG and to regulate humoral immunity by a negative feedback loop, which was found to reduce the generation of autoantibodies by binding to the Fc region of IgG ^[ 39] ^. Obexelimab, often referred to as XmAb5871, is an anti-CD19 and FcγRⅡb BsAb ( ***Fig. 1*** and ***Table 1***). This treatment inhibits B cell growth, calcium transport, and synthesis of co-stimulatory molecules in SLE patients; as a result, IgM, IgG, and IgE levels were reduced, and humoral immunity was inhibited ^[ 40] ^. In a phase Ⅱ clinical trial, B-cell counts were decreased by approximately 50% in 104 patients who received obexelimab, demonstrating the drug's safety and efficacy ^[ 41] ^.

### T cells and T cell co-stimulator blockers

T-cell dysfunction, particularly in T-helper cells, also known as CD4 ^+^ cells, plays a major role in the etiology of SLE ^[ 42] ^. Tfh has been shown to stimulate the differentiation of B lymphocytes in SLE patients and mouse models by producing interleukin (IL)-21 and forming autoreactive clones ^[ 43] ^. Th1 cells are predominant in SLE patients and have been shown to induce aberrant production of proinflammatory cytokines correlated with oxidative stress, including IL-12 and type Ⅰinterferon (IFN)-γ ^[ 44– 45] ^. On the other hand, peripheral blood from SLE patients was found to have fewer IL-4-producing Th2 cells, indicating a potential protective function and suggesting that SLE severity may be related to a higher IFN-γ/IL-4 ratio ^[ 46] ^. The primary source of IL-17, Th17 cells, is also implicated in the development of SLE. These Th17 cells have been shown to stimulate innate immunity, neutrophil recruitment, and B cell activation, as well as exacerbate tissue damage by inducing inflammation ^[ 47] ^. Further studies revealed that in lupus nephritis patients, IL-17 was correlated with the SLE disease activity index (SLEDAI) ^[ 48– 49] ^. A higher rate of Treg apoptosis was correlated with SLE severity, suggesting that CD4 ^+^ Treg cell abnormalities also affect immune tolerance in SLE patients ^[ 50] ^. It has been established that IL-2 levels and the IL-2/STAT5 signaling pathway control the quantity and functionality of CD4 ^+^ Tregs ^[ 51] ^. Recent studies demonstrated that treating patients with low doses of recombinant IL-2 was one of the ways to influence the course of SLE; such treatment led to the normalization of Treg cell functions and Tfr/Tfh balance, decreased formation of autoantibodies against dsDNA, and improved renal condition; furthermore, the restoration of the Tfr/Tfh ratio in SLE patients by the IL-2 therapy has been established and previously shown to be critical for maintaining immune tolerance and preventing autoantibody production ^[ 52– 53] ^. The possibility of regulating the activity of Treg cells will be further discussed in detail in the next section: "Therapeutic strategies for gut microbiota dysbiosis".

Immunological activation of B cells depends on the interaction of costimulatory signals with T cells, namely CD40/40L, CD28, CD80/CD86, and inducible T cell co-stimulator ligand. When CD40 on a B cell or antigen-presenting cell (APC) interacts with its ligand CD40L expressed on a T cell, CD80/CD86 and other co-stimulatory molecules are upregulated on the APC ^[ 54] ^. When CD28, which is constitutively expressed on T cells, binds to CD80/CD86 on an APC, the T cells become activated ( ***Fig. 1***). The activated T cell then begins to produce more cytotoxic T-lymphocyte-associated protein 4 (CTLA-4), which has a higher affinity for binding to CD80/CD86 than CD28 and inhibits T cell activation ^[ 55] ^. By blocking this pathway, it is possible to indirectly inhibit the development of B cells, their activation, and the production of antibodies, thereby producing a therapeutic effect ^[ 56] ^. The fusion protein abatacept, a soluble CTLA-4 analog, acts as an antagonist of CD28-mediated co-stimulation to prevent the specific interaction of CD28 with CD80/CD86, resulting in the inhibition of B-cell growth and activation ( ***Fig. 1***). Abatacept has demonstrated some efficacy and an acceptable safety profile in the treatment of SLE patients and is currently in phase Ⅲ clinical trials ( ***Table 1***) ^[ 57– 58] ^. Other inhibitors of T cell activity by blocking transmembrane proteins expressed by T cells, such as CD6, CD28, CD40, and CD74, have been developed and are currently in clinical trials ( ***Fig. 1*** and ***Table 1***).

### Targeting cytokines or cytokine receptors

It has also been demonstrated that type I interferon alpha (IFNα), primarily produced by plasmacytoid dendritic cells (pDCs), is critical in the etiology of SLE ^[ 59] ^. It has been shown that Toll-like receptor 7 (TLR7) and TLR9 expressed by pDCs regulate the IFNα activity ^[ 42] ^. By interacting with these receptors, IFNα increases the transcription of hundreds of genes involved in the autoimmune response ^[ 60– 61] ^. TLR7 and TLR9 were found to contribute to the synthesis of autoantibodies against RNA- and DNA-associated autoantigens in a murine model of SLE ^[ 62] ^. These findings suggest that targeting both TLRs and IFNα may be useful in the SLE treatment ^[ 63] ^. Phase Ⅲ clinical trials are currently underway for anifrolumab, an anti-IFNα mAb that has been approved by the FDA for the treatment of SLE in 2021 ( ***Fig. 1*** and ***Table 1***) ^[ 64] ^. Many new agents that target TLR7/8 have recently been developed and entered clinical trials, with the most promising one being the small molecule E6742 ^[ 63] ^. Future research on novel biological treatments that specifically target TLR7/8 is highly probable.

Inflammation and tissue damage in SLE are also caused by other cytokines: IL-6, IL-10, IL-12, and IL-18, and an increase in their concentration in the blood serum of patients is associated with an increase in the severity of the disease ^[ 65– 67] ^. Preventing cytokine activity with biological agents, many of which are in clinical trials, is another approach to the treatment of SLE ( ***Table 1***). Novel biological agents that inhibit cytokines, such as IL-6, IL-10, IL-12, IL-17, IFNɑ, TNFɑ, and IL21/IL23, have been developed in recent years and are undergoing clinical trials ( ***Fig. 1*** and ***Table 1***) ^[ 64] ^.

### Drugs targeting pDCs

There are at least two molecules that directly target pDCs. Litifilimab (BIIB059) is an IgG1 mAb that binds to blood dendritic cell antigen 2 (BDCA2), a cell surface receptor expressed exclusively by pDCs ( ***Fig. 1***) ^[ 68] ^. The treatment of SLE patients with litifilimab resulted in a significant reduction in the baseline level of inflamed joints, compared with the placebo for 24 weeks in phase Ⅱ clinical trials ( ***Table 1***). However, additional studies are needed to determine the safety and efficacy of litifilimab ^[ 69] ^. Litifilimab also showed some efficacy in a randomized phase Ⅱ clinical trial in patients with SLE using specific skin indicators, demonstrating the drug's promising potential for the treatment of this disease ^[ 70] ^. Daxdilimab (also known as VIB7734) is an anti-immunoglobulin-like transcript-7 mAb that specifically depletes pDCs. This antibody was designed to rapidly and efficiently destroy pDCs using the mechanism of antibody-dependent cell cytotoxicity. In a phase IA clinical trial, daxdilimab demonstrated a rapid depletion of pDCs in both cynomolgus monkeys and humans; furthermore, the reduction of pDCs in the skin was correlated with a decrease in local IFNα activity and improvement in other clinical indicators ^[ 71] ^.

### CAR-T cell therapy

The use of antibodies to treat SLE has several limitations. Two studies showed that the mAb rituximab, targeting CD20, did not completely deplete B cells in tissues; furthermore, a significant number of B cells survived depletion, thus highlighting the limitations of this method in some severe forms of the disease, despite significant advances in the treatment of SLE with antibodies ^[ 72– 73] ^. Another study showed that since plasma blasts and long-lived plasma cells did not express CD20, rituximab was ineffective in eliminating them, which was a factor in the formation of autoantibodies in lupus disease ^[ 74] ^. Based on these findings, targeting CD19 ^+^ B cells may provide some benefit, and thus it was decided to try anti-CD19 CAR-T cells for this purpose ( ***Fig. 1***). CAR-T cells are genetically modified T lymphocytes that carry a specialized receptor on their surface, which is necessary for recognition and subsequent binding to a specific antigen on the target cell ^[ 75] ^. Further studies showed that anti-CD19 CAR-T cells provided an additional advantage over anti-CD20 mAb therapy, because they might penetrate deep tissues; moreover, after isolation from peripheral blood T cells of the patient, CAR genes were inserted into the T cell genome to create CAR-T cells; and the developed CAR-T cells were then multiplied using modern bioreactor systems, analyzed, and reinfused back to the patient ^[ 76– 80] ^.

CAR-T cells, which are designed to target CD19 ^+^ B lymphocytes ( ***Fig. 1***), are now the most widely used to treat certain types of cancer ^[ 75] ^. Preclinical trials of CAR-T cells for SLE treatment were initially conducted in mice and demonstrated a reduction in autoimmune B cells and autoantibodies, as well as a relief of other symptoms ^[ 81– 82] ^. This therapy was later used to treat a patient with severe SLE, including nephritis, resulting in a sustained remission of the disease with the absence of anti-dsDNA autoantibodies ^[ 83] ^. Finally, a study involving five SLE patients showed similar results: a stable remission based on laboratory parameters, a significant decrease in B-cell counts, and the autoantibody formation were also achieved ^[ 11] ^. These studies suggest that the use of anti-CD19 CAR-T cells in SLE treatment is a promising, effective, and feasible approach.

### Benefits and drawbacks of treating SLE with antibodies and CAR-T cells

Despite obvious advantages and successes associated with the use of biologics and CAR-T cells for the treatment of autoimmune diseases, both methods have their drawbacks. Biologics offer more selective outcomes with fewer toxic effects than traditional treatments with glucocorticoids and immunosuppressants ^[ 84] ^. However, antibody treatments remain challenging, particularly in terms of determining the optimal dosage, which is usually ineffective, if the dose of antibodies is insufficient, while long-term administration of antibodies leads to immunogenicity ^[ 85] ^. Immunogenicity has several implications for the use of biologics. Repeated administration of recombinant homologs of certain human proteins, such as interferons or erythropoietin, especially in an aggregated or partially denatured form, has been shown to result in the production of anti-drug antibodies ^[ 84] ^. Anti-drug antibodies interfere with the bioavailability, pharmacokinetics, and efficacy of biological drugs, neutralizing their activity, and causing severe autoimmune, allergic reactions and other immune disorders ^[ 85] ^. Thus, an important component of antibody safety is the assessment of immunogenicity, especially when there is a concomitant immunosuppressive therapy that also affects the immune response ^[ 84] ^. The CAR T-cell therapy also has serious side effects, including potentially fatal cytokine release syndrome, which may occur in the treatment of cancer patients. However, the risk of cytokine release syndrome in SAIDs may be reduced, for example, by using appropriate antibodies that reduce the number of circulating B cells ^[ 86] ^.

Because antibody therapy does not require individualized development for each patient, it is more versatile and affordable than the CAR-T cell therapy ^[ 87] ^. Antibodies are also easier to dose and it is easier to adjust treatment regimens depending on the response of patients ^[ 88] ^. In addition, the ease of use, reproducibility of results, and scalability of production make antibodies more suitable for a large-scale use, compared with the CAR-T cell-based immunotherapies ^[ 89] ^. Although the CAR-T cell therapy is a safer and more effective treatment with promising prospects, it requires a complex and time-consuming manufacturing process; furthermore, another complication of the CAR-T cell therapy is the need for lymphodepletion before infusion ^[ 90] ^. Because the CAR-T therapy and lymphodepletion are resource-intensive treatments, they require more experienced personnel, manufacturing capacity, and hospital beds, and ultimately, significant funding ^[ 91] ^.

Based on the information outlined above, it may be beneficial to develop new, simpler, and less expensive methods of maintenance therapy for SLE without side effects in addition to immunotherapy. This may help to stabilize the patients, improve their quality of life, and increase their chances of survival. Approaches based on the pathophysiological role of the gut microbiota in the development of the disease may potentially become such new treatments.

## Therapeutic strategies for gut microbiota dysbiosis

### Alteration of the gut microbiota in SLE

In addition to environmental factors and genetic predisposition, the gut microbiota is believed to contribute to the development of SLE, although the pathogenesis of this disease is not well understood ^[ 14, 92] ^. A large number of studies indicate a link between the pathogenesis of SLE and various disorders of the gut microbiota. For example, one study sequenced fecal samples from 117 SLE patients and 115 healthy controls to investigate the gut microbiota in patients ^[ 93] ^. Compared with healthy controls, the gut microbiota of lupus patients had an autoimmune and pro-inflammatory profile, as several species were increased in the SLE gut microbiota but decreased after treatment, including the *Clostridium* species ATCC BAA-442, as well as *Actinomyces massiliensis*, *Atopobium rimae*, *Bacteroides fragilis*, *Shuttleworthia satelles*, and *Clostridium leptum*, suggesting that these species might contribute to lupus progression ^[ 93] ^. The general trend is that healthy individuals have a higher richness and diversity of gut microbiota than SLE patients, especially those with a higher SLEDAI score ^[ 94– 95] ^. Several studies demonstrated that a characteristic of the gut microbiota, such as the ratio of *Firmicutes* to *Bacteroidetes*, was lower in patients with SLE than in healthy individuals ^[ 95– 96] ^. The count of *Firmicutes* was correlated with the SLEDAI score, indicating that *Firmicutes* might slow the development of lupus ^[ 97] ^. There was also a significant decrease in *Bacteroidetes* abundance but an increase in potentially pathogenic *Proteobacteria* abundance in SLE patients ^[ 97] ^. In addition, *Ruminococcus gnavus* ( *R. gnavus*), a member of the *Lachnospiraceae* family, was found to be associated with disease activity, and was five times more abundant in the gut microbiota of SLE patients than in healthy controls; furthermore, anti-dsDNA antibody levels and SLEDAI score were found to be correlated with serum anti- *R. gnavus* antibody levels ^[ 94] ^.

The role of vaginal microbiota dysbiosis in SLE pathogenesis has also been investigated. One study examined fecal and vaginal samples from 30 SLE patients and 30 healthy controls to determine the specific composition of the gut and vaginal microbiome in patients with SLE disease ^[ 13] ^. The results showed that disease severity and immune system characteristics were correlated with changes in the gut and vaginal microbiota in SLE. Compared with healthy controls, SLE patients exhibited both fecal and vaginal dysbiosis, with the latter being more pronounced, and it appeared that only changes in the vaginal microbiota were correlated with changes in the immunological profile of patients ^[ 13] ^.

Characteristics of the gut microbiota in SLE mouse models have essentially supported conclusions from studies in SLE patients. The gut microbiota of SLE patients has been found to share characteristics with the gut microbiota of MRL/lpr mice, a classic mouse model for SLE research ^[ 93] ^. One study showed that the relocation of a gut pathogen, *Enterococcus gallinarum*, to the liver and other tissues in mice triggered autoimmune responses, suggesting that the impaired gut barrier function significantly influenced the progression of SLE ^[ 98] ^. Using the MRL/lpr mouse model, another study revealed that the increases in *Lachnospiraceae* in the gut microbiota were associated with lupus progression, and gut colonization with *Lactobacillaceae* was correlated with improved symptoms ^[ 99] ^. In addition, boosting the probiotic *Lactobacillus* spp. and eradicating the toxic bacteria *Lachnospiraceae* with antibiotic therapy in the MRL/lpr mouse model was found to reduce lupus symptoms ^[ 100] ^.

In summary, the changes in gut microbial composition associated with the onset and severity of SLE include a decrease in the number of beneficial bacteria, an overgrowth of potentially harmful bacteria, and a decrease in overall bacterial diversity. All of these changes indicate the involvement of intestinal dysbiosis in the pathophysiology of the disease.

### Potential mechanisms linking gut microbiota to SLE pathogenesis

One hypothesis describing a potential mechanism linking gut microbiota and SLE pathogenesis is that the impaired gut barrier function allows bacteria to migrate throughout the body to other tissues, triggering an immune response and resulting in autoantibody formation ^[ 101] ^. The intestine is well known to implement its barrier function at the physical, immunological, and biochemical levels to protect the host body from the aggressive effects of environmental factors ^[ 102] ^. A condition, in which microorganisms leak out of the intestinal lumen and migrate to other organs of the human body because of the reduced intestinal barrier function, is called "leaky gut", in which the impaired intestinal barrier function was found to increase the abundance of bacteria, such as *R. gnavus* and *E. gallinarum*, resulting in the release of inflammatory factors that aggravate systemic inflammation ^[ 103] ^. The bacterial translocation into the lamina propria with autoreactive T and B cells stimulates the toll-like pathway and the production of inflammatory cytokines, type Ⅰ IFN, and autoantibodies. These circulating inflammatory products lead to the loss of immune tolerance and organ damage.

The "leaky gut" syndrome also alters metabolic profile of the gut microbiota, which leads to a dysfunction of the immune system. Serum and fecal samples from SLE patients were found to contain several significantly altered gut microbiota metabolites, including pentanoate, short-chain fatty acids, glycolic acid, and bile acids, suggesting a link between lipid abnormalities and gut microbiota dysbiosis in SLE patients ^[ 97] ^. The metagenomic data also revealed that lipopolysaccharide production was increased and branched-chain amino acid biosynthesis was decreased in the gut of SLE patients ^[ 93] ^. Because of their anti-inflammatory properties, short-chain fatty acids (SCFAs) play an important role in the pathophysiology of lupus. These SCFAs potentially strengthen the intestinal barrier and mitigate autoimmunity by influencing innate lymphoid cells, T cells, DCs, and macrophages ^[ 104] ^. Under physiological conditions, the bacterial production of SCFAs ensures the normal differentiation of T and B lymphocytes ^[ 105] ^. *Firmicutes* bacteria are the main producers of butyrate, a member of the SCFA family that plays a central role in the generation and maintenance of Treg cells in the gut, which block the transdifferentiation of T cells into Th17 effectors and Th1 cells and ensure a balanced production of both anti-inflammatory and inflammatory cytokines ^[ 106] ^. A decreased *Firmicutes/Bacteroidetes* ratio in SLE resulted in a decreased biosynthesis of SCFAs, which causes immune dysregulation in SLE patients ^[ 107] ^. The *Ruminococcaceae* family has also been found to produce SCFAs, and a decrease in these bacteria may lead to a reduction in SCFA production ^[ 92] ^. Another metabolite of gut bacteria, tryptophan, also associated with the SLE severity, is metabolized differently in SLE patients, and may control the number of autoreactive helper T cells ^[ 12] ^. Dietary tryptophan was found to regulate the autoimmune system; a low dietary tryptophan prevented autoimmune disease in a mouse model of SLE, while a high dietary tryptophan exacerbated the disease ^[ 108] ^.

Autoantibodies may be formed by a special mechanism called molecular mimicry after bacteria and their components have entered the intestine. Molecular mimicry is a condition in which molecular structures of a microorganism resemble those of the host, causing an autoimmune response ^[ 109] ^. Bacteria with self-antigen-like epitopes have been suggested to induce the production of cross-reactive autoantibodies, thereby triggering the development of SLE ^[ 109] ^. In support of this hypothesis, there is evidence that Ro60 orthologs from human commensal bacteria may induce SLE autoimmunity and cross-react with human B cells and T cells ^[ 110] ^. In addition, molecular mimicry experiments identified several bacterial peptides derived from the SLE-enriched species that showed autoimmunogenic capacity ^[ 93] ^.

### Gut microbiota therapeutic approaches

**Figure 2:**
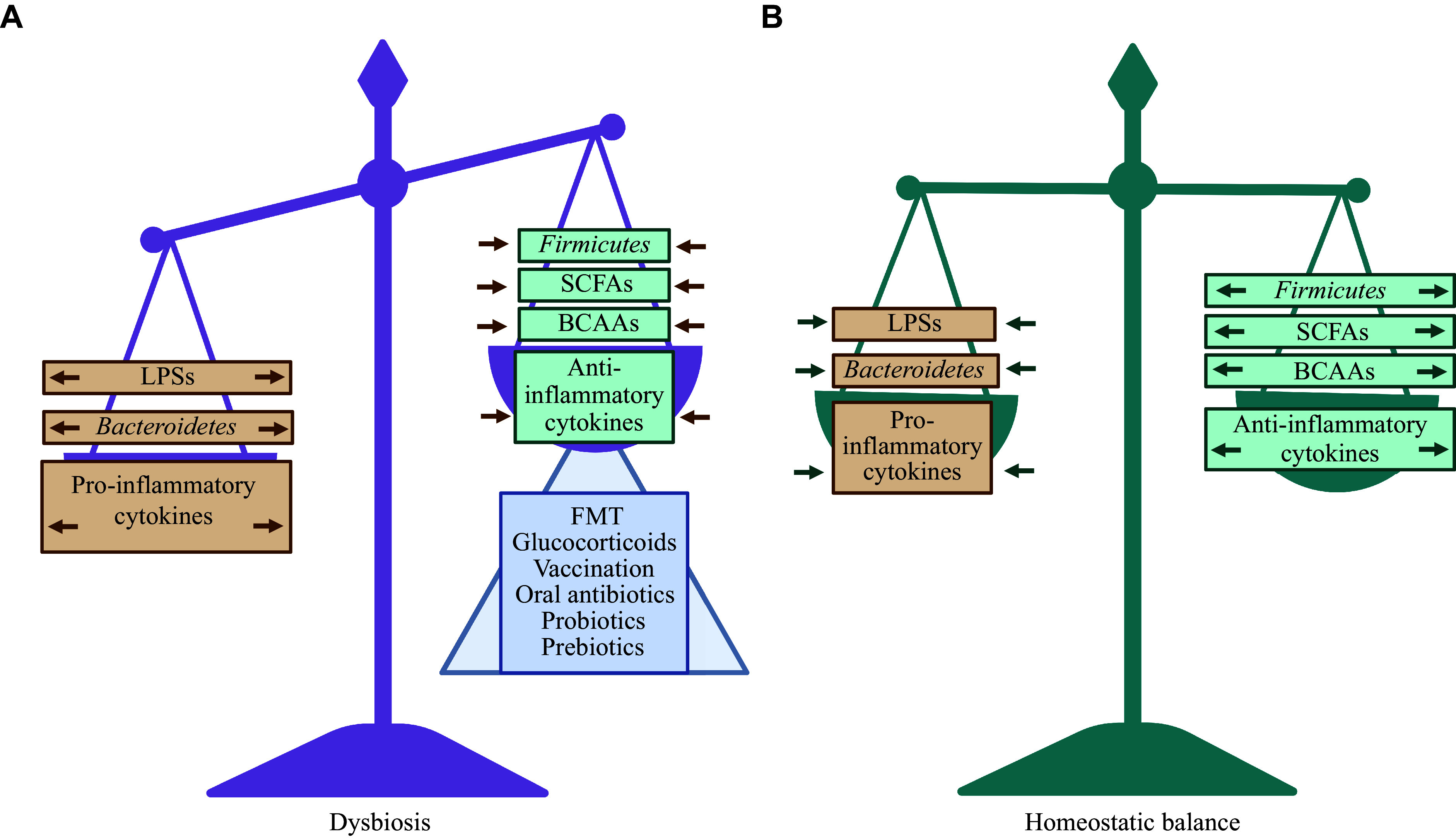
Potential pathophysiological mechanisms of gut microbiota therapy for systemic lupus erythematosus treatment. A: Gut microbiota dysbiosis is characterized by a decreased *Firmicutes*/ *Bacteroidetes* ratio, a reduced synthesis of SCFAs and BCAAs, lower levels of anti-inflammatory cytokines, and increased levels of LPS and pro-inflammatory cytokines. B: A healthy microbiome in a state of homeostatic balance. Ideally, after gut microbiota modulation procedures in SLE, including probiotic or prebiotic therapy, oral antibiotic therapy, glucocorticoid therapy, vaccination, and FMT, the imbalance should shift towards a homeostatic balance characterized by the restoration of the composition of microorganisms, the profile of metabolites and cytokines characteristic of a healthy body. Abbreviations: BCAAs, branched-chain amino acids; FMT, fecal microbiota transplantation; LPSs, lipopolysaccharides; SCFAs, short-chain fatty acids.

Several approaches may be used to correct gut microbiota dysbiosis in the treatment of SLE, each with the ultimate goal of shifting the bacterial composition, metabolite profiles, and host immune response toward a homeostatic balance that improves the patient's condition and contributes to the recovery ( ***Fig. 2***). The gut microbiota composition appears to be affected by glucocorticoid treatment in SLE patients. For example, the treatment of MRL/lpr mice with prednisone was found to alter the gut microbiota, including an increase in *Anaerostipes*, which has a clear relationship with the SLE severity, and a decrease in *Oscillospira*, *Mucispirillum*, *Rikenella*, and *Bilophila* ^[ 111] ^. In another study, mice had a significant decrease in *Lactobacillus* species that were associated with the SLE severity, compared with controls, with an increase in gut bacterial diversity after treatment with dexamethasone ^[ 112] ^. In addition, the use of glucocorticoids by the patients in the treatment of SLE has been found to increase the levels of probiotics, such as *Bifidobacterium* and *Lactobacillus*, and leads to the intestinal *Firmicutes*/ *Bacteroidetes* ratio typical of healthy people ^[ 113] ^. All these data suggest that glucocorticoids may normalize the gut microbiota in SLE patients.

Some vaccines against specific pathogenic bacteria, such as *E. gallinarum* and others, have also been shown to help treat SLE. For example, vaccination against *E. gallinarum* in lupus (NZW × BXSB) F1 mice was found to reduce the progression of SLE and restore intestinal barrier function ^[ 98] ^. Considering the presence of *E. gallinarum* not only in the intestine but also in the liver, vaccination against this bacterium may be an effective approach to the treatment of SLE patients after necessary improvements have been made ^[ 98] ^.

According to several studies in a mouse model of SLE, antibiotics may also be an available treatment option. For example, the treatment of lupus mice with vancomycin or ampicillin was found to eradicate *E. gallinarum*, a specific intestinal pathogen, delayed lupus progression, improved intestinal barrier function, and prevented mortality ^[ 98] ^. The treatment of SLE with vancomycin or other antibiotics in the MRL/lpr mice has also been shown to eradicate harmful gut bacteria *Lachnospiraceae* and enrich some probiotics, such as *Lactobacillus* spp., resulting in the restoration of gut barrier function, amelioration of lupus and alleviation of the Treg/Th17 imbalance ^[ 100] ^. However, there are data demonstrating that the use of antibiotics in the NZB/WF1 lupus mice has no effect on autoantibody production and SLE severity, nor the composition of the gut microbiota, suggesting that further studies are needed to determine the effectiveness of antibiotics in treating SLE ^[ 114] ^. Antibiotic treatment was also shown to be dose-dependent. Short courses and inadequate doses of antibiotics before the onset of lupus exacerbated SLE activity by depleting beneficial gut microbiota, such as *Bifidobacterium* and *Lactobacillus*, and enriching harmful bacteria, such as *Proteus* and *Klebsiella* in the MRL/lpr mice ^[ 115] ^. According to another study, the treatment of MRL/lpr mice with vancomycin reduced the severity of SLE in normal mice but worsened the disease in pregnant and postpartum mice by suppressing Treg expression, suggesting that the use of antibiotics may have adverse effects ^[ 116] ^.

Another method of correcting the gut microbiota is the use of probiotics or prebiotics. Probiotics, particularly *Lactobacillus* and *Bifidobacteria* strains, help control the inflammatory state and reduce autoantibody production and the SLE severity ^[ 103, 117] ^. For example, *Bifidobacterium* has been shown to maintain the balance of Treg, Th17, and Th1 cells by preventing excessive activation of CD4 ^+^ T cells in SLE patients ^[ 118] ^. By lowering anti-dsDNA levels, *Lactobacillus* spp. supplementation in the MRL/lpr mice showed a significant effect in preventing lupus nephritis and prolonging mouse life ^[ 119– 120] ^. Another study using the MRL/lpr mouse model demonstrated the feasibility of using a strain of *L. casei shirota* obtained from a healthy donor as a probiotic; furthermore, this bacterial strain was found to have immunomodulatory properties, promoting macrophage infiltration without compromising T-cell activity and ultimately prolonging the life of MRL/lpr mice ^[ 119] ^. In addition, *L. delbrueckii* subsp. lactis (PTCC 1743) and *L. rhamnosus* (ATCC 9595) improved disease symptoms in a pristane-induced lupus mouse model and reduced Th17 cell populations and IL-17a levels ^[ 121] ^.

One of the most powerful approaches to restoring the microbiota balance is FMT. FMT is defined as the injection of a fecal suspension obtained from a healthy donor into the gastrointestinal tract of a patient to restore a stable gut microbiota ^[ 122] ^. The therapeutic potential of this procedure was first studied in intestinal infections, and 10 years ago, FMT was added to the standard treatment recommendations for the *Clostridium difficile* infection ^[ 123] ^. Recent studies have confirmed the efficacy of FMT not only for intestinal infections but also for the treatment of SLE, initially in mouse models ^[ 124– 125] ^. FMT was observed to improve antibiotic-induced gut microbiota dysbiosis one week after antibiotic exposure and reduce the severity of lupus in the MRL/lpr mice ^[ 115] ^. One study also showed that untreated mice might be transplanted with the fecal microbiota of prednisone-treated mice without side effects ^[ 124] ^. Clinical trials of FMT have already been conducted in patients with other autoimmune diseases, such as type 1 diabetes and ulcerative colitis, with some success ^[ 126– 127] ^. FMT was applied to a patient with SLE who was infected with a *Blastocystis hominis* parasite and who also had glomerulonephritis, malnutrition, diarrhea, and severe weight loss; after the FMT treatment, there was an improvement in diarrhea and anxiety ^[ 128] ^. Another 20 patients with active SLE were recruited to participate in a 12-week pilot clinical trial of orally encapsulated FMT; after the FMT therapy, both serum anti-dsDNA antibody levels and the SLEDAI score were significantly reduced; furthermore, in addition to an increased synthesis of SCFAs in the gut and decreased levels of IL-6 and the CD4 ^+^ memory/naive ratio in peripheral blood, there was an enrichment of beneficial SCFA-producing bacterial taxa and a reduction in inflammation-related bacterial taxa; no deaths or serious adverse events were reported ^[ 15] ^. These data suggest that FMT appears to be a realistic, safe, and potentially successful option for the treatment of SLE ^[ 15– 16] ^. Thus, the use of FMT for the treatment of SLE is a promising approach that deserves to be confirmed in future studies.

Although FMT has been shown to be safe and free of side effects in clinical trials, there are some limitations to its use. Lupus patients are thought to be more susceptible to latent pathogens because of prolonged use of immunosuppressive drugs, and this poses unknown risks. Therefore, to prevent the transmission of pathogenic bacteria, viruses, and fungi from the donor to the recipient, a series of medical examinations are performed on the donor, including a detailed medical history as well as blood and stool tests ^[ 16] ^. Donors with infectious diseases, autoimmune diseases, gastrointestinal diseases, metabolic syndrome, surgery, or those taking antibiotics or immunosuppressants are not eligible ^[ 129] ^. Evaluation of the recipient is equally important. General contraindications include pregnancy, infectious diseases ( *e.g.* tuberculosis, hepatitis B, and HIV), recent gastrointestinal surgery, anticoagulant therapy, and use of antibiotics or probiotics. The FMT therapy is strongly discouraged in SLE patients with severe organic lesions, such as proteinuria and lupus encephalopathy ^[ 16] ^.

## Conclusions and perspectives

Despite significant advances in the treatment of SLE, some patients are at high risk of organ failure and even death, because they do not respond to the current standard of care. As a result, new alternatives have emerged, such as immunotherapy. Molecular targets for the antibody therapy include B cells, T cells or T cell co-stimulator blocks, and pro-inflammatory cytokines or cytokine receptors. There are now clinical trials for certain biologic drugs that have shown some effective activity in SLE patients. In addition, the generation of CAR-T cells offers a novel way to overcome some of the drawbacks of antibody therapy, such as immunogenicity, side effects from repeated injections, and incomplete autoantibody depletion. Recently, SLE patients have been effectively treated with anti-CD19 CAR-T cells, paving the way for the treatment of other SAIDs, and the progress of this treatment has produced positive results. However, even CAR T-cell treatments have limitations related to the high cost and complexity of the procedure. Improving the specificity and safety, reducing the immunogenicity of antibodies and other biological agents, improving the safety and reducing the cost of CAR-T therapy, are important goals for future studies.

Recently, investigators have focused on the possibility of alleviating patient conditions by correcting dysbiosis of the intestinal microbiota. Although the correlation between gut dysbiosis and immune dysregulation in SLE has not been established, it is not clear whether gut microbiome dysbiosis is a consequence of the disease or a driving factor in its pathogenesis; however, there is at least a strong correlation between these processes. Some recent evidence shows that the gut microbiota has a systemic effect on the development and function of the immune system, thus affecting autoimmunity. Based on the available data, the proposed interaction between gut microbiota and systemic immunity in the pathogenesis of SLE may be as follows. In a healthy body, the intestinal barrier is intact, the microbiota is diverse, and the increased *Firmicutes/Bacteroidetes* ratio provides levels of SCFAs that promote normal B and T cell differentiation, Treg cell regulation, and the production of anti-inflammatory cytokines. The condition of intestinal dysbiosis is accompanied by a decrease in the *Firmicutes/Bacteroidetes* ratio and a decrease in the biosynthesis of SCFAs, leading to the overactivation of Treg cells and Th17 transdifferentiation. In the leaky gut syndrome, the translocation of pathogenic bacteria, such as *R. gnavus* and *E. gallinarum*, into the lamina propria increases their antigen exposure and leads to the production of various proinflammatory cytokines, including IFNα, IL-6, and IL-17, as well as autoantibodies. These circulating inflammatory products contribute to an exaggerated immune response, a loss of auto tolerance, as well as tissue and organ damage. However, this process may be reversible, and correcting gut dysbiosis toward a healthy pattern has been shown to improve SLE symptoms. Targeting the gut microbiota also offers a considerable potential for an effective SLE therapy, especially because it does not require expensive drugs or equipment, but only the mobilization of the body's internal resources. Based on the above, the potential pathophysiological mechanism of gut microbiota therapy for SLE treatment is to restore the composition of bacteria inherent in a healthy body and to restore the bacterial metabolic profile. This appears to be precisely what is related to the therapeutic effect.

Gut microbiota balance may be controlled by using glucocorticoids, antibiotics, probiotics or prebiotics, vaccination, and FMT. Although oral antibiotics have some efficacy, their use is still controversial, because they may also worsen gut dysbiosis or cause the emergence of drug-resistant bacteria. Probiotics and prebiotics have shown some promise in previous studies for treating SLE by modulating the gut microbiota, but their effectiveness has not yet been proven in clinical trials, and more research is needed. Although not currently available for treatment in humans, vaccination against gut pathogenic bacteria slows down the progression of lupus in lupus mice without the use of antibiotics. By restoring the balance of gut bacteria and the function of the intestinal barrier, FMT has been reported to ameliorate disease in lupus mouse models. Clinical trials of FMT in SLE patients have also shown encouraging results. The FMT therapy has shown a high level of safety with no serious side effects except for transient gastrointestinal symptoms, although a medical evaluation is required before the procedure because of strict contraindications for both fecal donors and patients. In summary, the available evidence suggests that patients with SLE may benefit from adjuvant therapies that modify the gut microbiota, particularly FMT, as the procedure is effective and safe.

Healthcare providers may improve the outcomes of SLE and treat symptoms more effectively by understanding the unique characteristics of each therapeutic agent. Immunotherapy has a promising future in precision medicine, with a significant potential to improve the treatment of SAIDs and patient outcomes. Although the interaction between gut dysbiosis and immune dysfunction currently appears to be complex and the causal correlation between them is not clear, initial steps have already been taken in using gut microbiota therapy for the treatment of SLE, showing the prospects for development in this field. Future studies investigating the gut microbiota imbalance are needed, and a comprehensive understanding of its mechanisms may lead to innovative and effective therapeutics for the treatment of SLE, in addition to immunotherapy and other approaches.
